# Effect of DNA Methylation Modulators on UV Damage Formation and Repair  

**DOI:** 10.3390/genes17040487

**Published:** 2026-04-19

**Authors:** Kyle Jones, Rishav Rajbhandari, Wentao Li

**Affiliations:** Department of Environmental Health Science, College of Public Health, University of Georgia, Athens, GA 30602, USA

**Keywords:** DNA methylation, SAM, RG108, UV damage, nucleotide excision repair

## Abstract

**Background/Objectives:** DNA methylation is a key epigenetic modification involved in regulating many cellular processes, including gene expression and the maintenance of genome stability. Ultraviolet (UV) radiation induces DNA damage in the form of pyrimidine-pyrimidone (6-4) photoproducts [(6-4)PPs] and cyclobutane pyrimidine dimers (CPDs), which can lead to mutations if not efficiently repaired. While cytosine methylation has been implicated in influencing UV-induced DNA damage formation, the effect of DNA methylation modulators such as S-adenosyl-L-methionine (SAM) and RG108 on UV damage formation and repair remains unclear. **Methods:** Here, using immunoslot blot assays, we investigated the effects of SAM and RG108 on UV-induced DNA damage formation and repair in human lymphoblastoid cells. **Results:** We found that SAM, but not RG108, rapidly suppresses the formation of both (6-4)PP and CPD, with detectable effects within minutes of exposure. Although SAM pretreatment was associated with modestly accelerated early (6-4)PP repair, this effect was accompanied by substantially lower initial damage levels. When cells were treated with SAM or RG108 immediately after UV irradiation to ensure equivalent initial damage burden, no significant differences in repair were observed for either lesion type, demonstrating that the accelerated early (6-4)PP repair reflects reduced lesion burden rather than increased intrinsic nucleotide excision repair (NER). Global 5-methylcytosine (5mC) levels remained stable following SAM or RG108 treatment and during UV damage repair, suggesting that these effects occur independently of global alterations in DNA methylation. **Conclusions:** Together, our findings reveal that SAM modulates UV damage susceptibility at the level of lesion formation without altering repair, highlighting a previously unrecognized role for DNA methylation modulators in regulating genome stability.

## 1. Introduction

DNA methylation is a fundamental epigenetic mechanism involved in regulating gene expression, organizing chromatin, and maintaining genome stability without altering the DNA sequence [1,2,3]. In vertebrate genomes, approximately 70–80% of CpG dinucleotides are methylated, highlighting the pervasiveness of this modification [4]. DNA methylation contributes to transcriptional repression through multiple mechanisms, including blocking transcription factor binding and recruiting methyl-CpG-binding proteins that promote chromatin compaction and gene silencing [5,6,7]. Conversely, dysregulation of DNA methylation is strongly associated with tumorigenesis, aging-related diseases, and genomic instability [8,9,10]. DNA methylation patterns are established and maintained by DNA methyltransferases (DNMTs), where DNMT3A and DNMT3B function as de novo methyltransferases and DNMT1 maintains methylation patterns during DNA replication [11,12]. The universal methyl donor S-adenosyl-L-methionine (SAM) provides the methyl groups used by DNMTs and serves as a central metabolite linking one-carbon metabolism to epigenetic regulation [13]. Alterations in cellular SAM levels can influence global DNA methylation and transcriptional programs, and SAM supplementation has demonstrated anticancer effects through epigenetic silencing of oncogenes and modulation of tumor suppressor pathways [14,15]. Importantly, DNA methylation remains dynamically reversible through TET-mediated oxidation of 5-methylcytosine (5mC) followed by thymine DNA glycosylase (TDG)-initiated base excision repair (BER), enabling rapid adaptation of epigenetic states in response to cellular and environmental signals [16,17].

UV irradiation induces DNA photolesions through chemical reactions between two adjacent pyrimidines on the same DNA strand. The two major lesions are cyclobutane pyrimidine dimers (CPDs) and pyrimidine-pyrimidone (6-4) photoproducts [(6-4)PPs]. CPDs arise through a linkage involving the C5 and C6 positions of two neighboring pyrimidines, forming a cyclobutane ring. In contrast, (6-4)PPs form through a covalent bond between the C6 position of one pyrimidine and the C4 position of the adjacent pyrimidine [18,19]. These photolesions are initiated by UV-induced excitation of DNA bases through direct photon absorption by DNA. However, recent evidence demonstrates that delayed or ‘dark’ CPDs can arise after UV exposure through chemiexcitation-driven processes, particularly in melanin-containing cells [20,21]. CPDs represent the most abundant UV-induced photoproducts, accounting for approximately 75% of lesions following UV exposure, while (6-4)PPs occur at a lower frequency, comprising nearly 25% of total photolesions [22], and yet induce a stronger helix distortion than CPDs. Due to their stronger helix distortion properties, they can be recognized by nucleotide excision repair (NER) more easily than CPDs. The formation of UV photolesions is also influenced by several factors, including DNA sequence context, DNA methylation, and chromatin accessibility, which can either promote or suppress photolesion formation [23,24,25]. In regions of high susceptibility, the formation of multiple nearby photolesions may contribute to local clustering of damage. Tandem DNA lesions are defined as two contiguous damaged nucleotides generated by a single damaging event, often arising from radical-mediated reactions in which a reactive intermediate on one nucleotide reacts with an adjacent nucleotide. Such lesions represent a subset of clustered DNA damage and can significantly impact DNA repair efficiency by challenging canonical repair pathways [26]. These lesions block cellular processes such as DNA replication and transcription by preventing polymerase progression along the damaged template strand. If not repaired efficiently, UV lesions can ultimately lead to frequent C>T transitions as well as CC>TT double substitutions, representing a characteristic UV mutational signature frequently found in melanomas [27,28]. These mutations can occur in key driver genes, including *TERT*, *BRAF*, and *NRAS*, which are frequently altered in melanoma [29,30]. Current standard therapeutic strategies include surgery, radiotherapy, and chemotherapy. However, these methods are limited against advanced or metastatic melanomas and are associated with poor clinical outcomes [31]. More recent treatment strategies include immunotherapy (e.g., immune checkpoint inhibitors blocking PD-1 and CTLA-4) and targeted therapies that inhibit key oncogenic signaling pathways (e.g., inhibitors against the BRAF and MEK) [31,32]. Recent studies have further highlighted emerging therapeutic strategies, including blocking nitrosylation to sensitize NRAS-mutant melanoma to MEK inhibition and to enhance antitumor immune responses [33].

To remove the helix-distorting UV lesions, cells use the NER pathway, a versatile repair mechanism responsible for removing a variety of bulky and helix-distorting DNA lesions. In addition to UV-induced DNA damage, NER also eliminates lesions caused by environmental carcinogens, including aflatoxin, acetaldehyde, and benzo[a]pyrene [34,35,36,37]. NER operates through two subpathways: global genome repair (GGR) and transcription-coupled repair (TCR). They are distinguished by how the lesion is recognized. In GGR, XPC, along with UV-DDB complex, surveys the entire genome for structural distortions, while TCR is triggered when RNA polymerase II (RNAP II) stalls at a DNA lesion during transcription [38,39,40,41,42]. Recent studies have identified the protein STK19 as an important factor that facilitates recruitment and positioning of TFIIH at sites of transcription-blocking lesions, promoting efficient handoff from stalled transcription complexes to the NER machinery [43,44,45,46,47]. Once TFIIH is properly positioned, the downstream excision and DNA synthesis steps proceed similarly to those in GGR. Together, these subpathways enable cells to rapidly remove most UV damage. While BER can repair DNA methylation, it is the primary pathway for repairing non-helix-distorting DNA lesions caused by endogenous sources of DNA damage, such as oxidation, alkylation, and deamination [48,49,50].

Previous studies have investigated the effects of DNA methylation on UV damage formation [23,51,52,53]. Early work by Tommasi et al. [23] demonstrated that adding SAM increased CPD formation within the p53 gene following UVB and solar radiation, while UVC had no measurable effect at dipyrimidine sites. Mitchell [53] reported that CPDs formed approximately twofold more frequently at methylated cytosines than at unmethylated cytosines following UVB treatment, whereas UVC treatment showed no methylation-dependent difference. In contrast, (6-4)PPs were found to increase at 5mC sites when treated with either UVB or UVC. Rochette et al. [54] examined naturally hypermethylated regions, including the inactive X chromosome and the methylated FMR1 locus in fragile X syndrome, and also found that UVB, but not UVC, induced significantly higher CPD formation at methylated sites compared to unmethylated regions. Leung et al. [52] showed that the effects of cytosine methylation on CPD and (6-4)PP formation are sequence-dependent, showing that many 5mC sites had a reduction in damage formation under both UVB and UVC. A follow-up study from Wilson and Wyrick [24] used CPD-seq to show that UVB increases CPD formation at methylated cytosines, whereas UVC effects are sequence-dependent, increasing damage at some motifs (e.g., GTCG, CCCG) while reducing it at others (e.g., TTCG, CTCG). Together, these findings highlight that DNA methylation, local nucleotide sequence, and UV wavelength play key roles in determining UV lesion formation. Meanwhile, the deamination of methylated cytosines within CPDs generates C>T transition mutations that strongly contribute to melanoma mutational signatures [55,56,57]. Thus, DNA methylation not only influences UV damage formation but also shapes its downstream mutational consequences.

SAM, the second-most used enzyme substrate in cellular metabolism, is widely available as a nutritional supplement for promoting mental health and joint function [58]. It has also been shown to suppress the growth and invasiveness of certain cancer cell lines, making it an attractive anticancer agent [14]. In contrast, RG108 is a non-nucleoside DNMT inhibitor that binds to the active site of DNMT to reduce DNA methylation levels with low toxicity. It has shown potential for reactivating epigenetically silenced tumor suppressor genes in cancer cells [59]. While the status of DNA methylation has been shown to influence UV damage formation, the effect of DNA methylation modulators, SAM and RG108, on UV-induced DNA damage formation and subsequent repair remains largely unexplored. In this study, we quantified UV-induced DNA damage formation and time-resolved repair rates to determine whether these two DNA methylation modulators affect damage susceptibility and repair efficiency in human lymphoblastoid cells. Our results reveal that SAM, but not RG108, rapidly suppresses UVC-induced (6-4)PP and CPD formation. We further observed an increase in (6-4)PP repair following SAM treatment compared to RG108-treated and control cells. However, this accelerated (6-4)PP repair rate reflects the relatively low initial burden of (6-4)PPs rather than intrinsically enhanced NER activity. Together, our findings reveal previously unrecognized roles for these two DNA methylation modulators in regulating UV damage susceptibility and repair dynamics.

## 2. Materials and Methods

### 2.1. Cell Culture

GM12878 human B-lymphoblastoid cell line was purchased from the National Institute of General Medical Sciences Human Genetic Cell Repository (Coriell) and was cultured in Roswell Park Memorial Institute (RPMI) 1640 medium with no phenol red, supplemented with 10% FBS and 1% penicillin/streptomycin at 37 °C in a 5% CO_2_ humidified chamber. Phenol red-free medium was used to avoid interference with UVC irradiation.

### 2.2. Drug Treatments

SAM-1,4-butanedisulfonate (D622436) was acquired from eNovation Chemicals (Bridgewater, NJ, USA), and RG108 (QI-8586) was obtained from Combi-Blocks (San Diego, CA, USA). A 100 mM SAM stock solution was prepared by dissolving 10 mg of SAM-1,4-butanedisulfonate in 138 µL of water and subsequently diluted 1:100 to obtain a final working concentration of 1 mM. A 10 mM RG108 stock solution was prepared by dissolving 1 mg of RG108 in 300 µL of DMSO and diluted 1:1000 to achieve a final concentration of 10 µM for treatment. Cells were treated with freshly made SAM or RG108 for specific durations before or after UVC irradiation. Previous studies have demonstrated that SAM and RG108 are stable in aqueous conditions over the time frames used in cell-based assays [60,61]. Untreated cells were included as controls in all experiments.

### 2.3. UV Irradiation

Cells were treated with the respective drugs and exposed to 254-nm UV light for either 5 s (10 J/m^2^) for (6-4)PP or 2.5 s (5 J/m^2^) for CPD in repair experiments, or 5 s (10 J/m^2^) for damage-only assays. At each repair time point, one-quarter of the cells were collected, while the remaining cells were further incubated in the CO_2_ incubator. Cells were then washed twice with ice-cold PBS and collected by centrifugation at 300 *g*. Cells without UV irradiation were included as controls.

### 2.4. Antibodies

The following antibodies were used in this study: primary antibodies, including anti-(6-4)PP (NM-DND-002) and anti-CPD (NM-DND-001), were purchased from Cosmo Bio (Tokyo, Japan), and anti-5-methylcytosine (D3S2Z) was purchased from Cell Signaling (Danvers, MA, USA). Secondary antibodies, rabbit anti-mouse-HRP-linked IgG (7076S) and goat anti-rabbit-HRP IgG (7074P2), were purchased from Cell Signaling.

### 2.5. Immunoslot Blot

Repair of UV-induced lesions was carried out as previously described [62], except that non-adherent cells were used instead. Cells were irradiated with UVC and allowed to repair for specific durations. Following incubation, cells were collected and washed with ice-cold PBS. DNA was isolated using the QIAgen Mini kit (Qiagen #51306, Hilden, Germany), and DNA concentrations were quantified using a Qubit. DNA [150 ng for (6-4)PP or 80 ng for CPD] was loaded onto a Bio-Dot SF apparatus (Bio-Rad, Hercules, CA, USA) and bound to a nitrocellulose membrane. The membrane was then incubated in a vacuum drying oven at 80 °C for 90 min. The membrane was blocked in 5% milk and incubated with either damage-specific primary antibodies, followed by incubation with the corresponding secondary antibodies. After incubation, enhanced chemiluminescence (ECL) was used for imaging using a Bio-Rad ChemiDoc Imaging System. Membranes were subsequently stained with SYBR Gold to assess DNA loading. Samples from no-UV or no-drug-treatment groups were included in all experiments. Immunoslot blot experiments were performed in triplicate.

### 2.6. Statistical Analysis

Statistical Analysis was done as previously described [63]. Ratios of damage intensities over DNA intensity were obtained. These ratios were normalized either to the 0 h time point for repair assays or to the untreated control group for damage formation assays. Triplicates were carried out for each experiment. Results were analyzed using two-way ANOVA followed by multiple-comparison testing in Prism v6 (GraphPad Software, San Diego, CA, USA). Data are presented as mean ± SEM, with statistical significance defined as (* *p* ≤ 0.05; ** *p* ≤ 0.01; *** *p* ≤ 0.001; **** *p* ≤ 0.0001).

## 3. Results

### 3.1. SAM, but Not RG108, Rapidly Suppresses UVC-Induced (6-4)PP and CPD Formation

To determine whether altered DNA methylation states influence UV lesion formation, we treated cells with SAM or RG108 prior to UVC irradiation. We then quantified the damage formation levels of the two UV photoproducts, (6-4)PPs and CPDs, following different treatment durations and concentrations. These complementary approaches allowed us to assess whether modulation of DNA methylation affects the overall UV damage burden. In our initial experiments, GM12878 cells were pretreated with 1 mM SAM or 10 µM RG108 for 6, 12, and 24 h before UV irradiation. SAM treatments consistently decreased the formation of both (6-4)PPs and CPDs. In contrast, RG108 treatment did not produce significant changes in UV damage formationdata not shown. This consistent suppression of UV damage formation following SAM treatment prompted us to conduct a further investigation into the temporal dynamics of this effect to determine how rapidly SAM induces this suppression.

**Figure 1:**
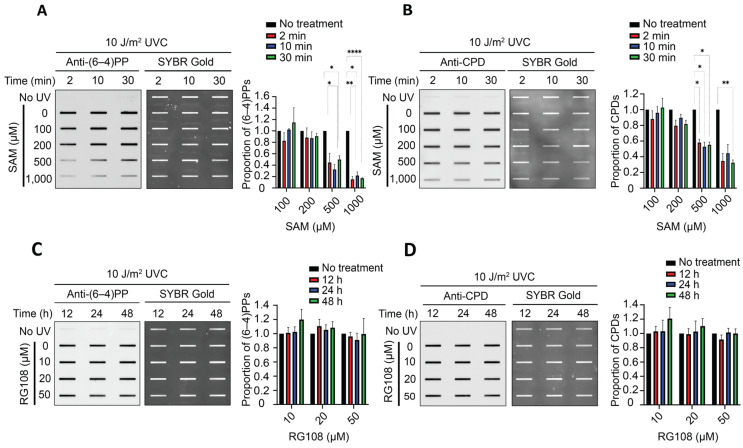
**SAM rapidly suppresses both (6-4)PP and CPD formation, while RG108 has no significant effect on UV damage formation.** (**A**) Representative immunoslot blot and quantification of (6-4)PP formation in GM12878 cells measured immediately after UVC irradiation (10 J/m^2^) following SAM pretreatment (2, 10, or 30 min) at different concentrations (0, 100, 200, 500, or 1000 µM). (**B**) Representative immunoslot blot and quantification of CPD formation from the same DNA samples shown in (**A**) using anti-CPD antibody. (**C**) Representative immunoslot blot and quantification of (6-4)PP formation measured immediately after UVC irradiation (10 J/m^2^), following UVC irradiation (10 J/m^2^) after RG108 pretreatment (12, 24, or 48 h) at varying concentrations (0, 10, 20, or 50 µM). (**D**) Representative immunoslot blot and quantification of CPD damage formation from the same DNA samples shown in (**C**) using anti-CPD antibody. All quantified damage levels are shown relative to untreated controls. Results shown are the mean ± SEM from three biological replicates. Statistical analysis was performed using two-way ANOVA, followed by multiple-comparison testing. Significance is indicated as: * *p* ≤ 0.05; ** *p* ≤ 0.01; **** *p* ≤ 0.0001.

To address this, we shortened the SAM treatment durations to 2, 10, and 30 min prior to irradiation. Remarkably, we observed that both (6-4)PP and CPD formation were reduced after only 2 min of SAM exposure, with no further substantial decrease at longer time intervals (Figure 1A,B). These findings indicate that SAM rapidly suppresses UVC-induced DNA damage formation within minutes of treatment. In contrast, RG108 treatment did not consistently alter the levels of either (6-4)PPs or CPDs across different concentrations or incubation times (Figure 1C,D). Although we hypothesized that DNMT inhibition by RG108 would decrease global DNA methylation and produce an effect opposite to that of SAM, we observed that RG108-treated cells exhibited UV damage levels comparable to untreated controls, even after 48 h of RG108 exposure, with no statistically significant differences.

Together, these results demonstrate that SAM, but not RG108, rapidly reduces UV-induced DNA damage formation. Notably, this effect was detectable within minutes at higher SAM concentrations. This rapid onset suggests that the suppression mechanism is independent of transcriptional reprogramming and likely occurs through an immediate chromatin-level change, such as direct modification of histone tails by SAM.

### 3.2. SAM Modestly Accelerates Early (6-4)PP Removal but Does Not Alter CPD Repair

**Figure 2:**
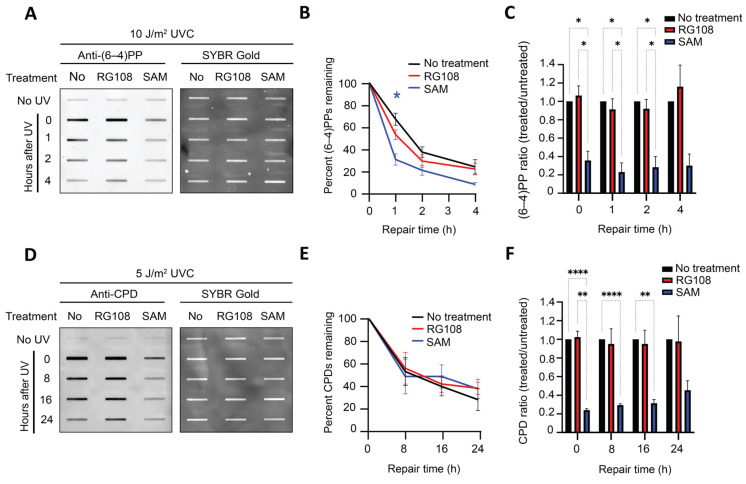
**SAM treatment prior to UVC irradiation is associated with accelerated early (6-4)PP repair but does not alter CPD repair**. (**A**–**C**) Immunoslot blot assays showing (6-4)PP damage levels, repair kinetics, and (6-4)PP damage ratio (treated/untreated) within 4 h in GM12878 cells treated with SAM (1000 µM) or RG108 (10 µM) for 24 h before UVC irradiation (10 J/m^2^). (**D**–**F**) CPD damage levels, repair kinetics, and CPD damage ratio (treated/untreated) within 24 h in GM12878 cells were analyzed and presented as in (**A**–**C**), except that an anti-CPD antibody was used. Data shown are the mean ± SEM from three biological replicates. Statistical analysis was performed using two-way ANOVA followed by multiple comparisons. Significance is indicated as: * *p* ≤ 0.05; ** *p* ≤ 0.01; **** *p* ≤ 0.0001.

Having established that SAM, but not RG108, suppresses global UV damage formation, we next sought to examine whether these two DNA methylation modulators affect NER. GM12878 cells were pretreated with SAM (1000 µM) or RG108 (10 µM) for 24 h prior to UVC irradiation, and repair kinetics were quantified as the percentage of remaining damage at each time point relative to the initial damage level at 0 h. Analysis of (6-4)PP repair revealed a modest but statistically significant decrease in the percentage of remaining (6-4)PP at 1 h in SAM-treated cells compared to untreated controls, indicating accelerated (6-4)PP repair at this early time point. Although RG108-treated cells also showed a modest trend toward faster (6-4)PP repair within 2 h, this effect did not reach statistical significance (Figure 2A,B). As expected, SAM-treated cells also exhibited substantially lower initial lesion formation at the 0 h time point (Figure 2C), consistent with the above SAM-mediated suppression of UV damage formation. For CPD repair, neither SAM nor RG108 produced a significant change in repair kinetics across all time points (Figure 2D,E), indicating that altered DNA methylation does not measurably impact CPD repair. However, SAM pretreatment significantly decreased the initial CPD level at 0 h (Figure 2F), supporting an effect on CPD formation rather than CPD repair.

These results indicate that SAM reduces the initial levels of both (6-4)PP and CPD. An acceleration of early (6-4)PP removal was observed, and CPD repair remained unaffected in SAM-treated cells. In contrast, RG108 did not significantly alter either UV lesion formation or removal of (6-4)PPs and CPDs.

### 3.3. Global 5mC Levels Remain Stable After SAM or RG108 Treatment and During UV Damage Repair

Given that SAM serves as a methyl donor and RG108 inhibits DNMT to reduce DNA methylation levels, we next asked whether the alterations in UV damage formation and repair kinetics were accompanied by changes in global DNA methylation levels. If the effects of SAM or RG108 on UV damage formation and repair were mediated through changes in cytosine methylation, corresponding shifts in global 5mC levels would be expected. Conversely, the absence of detectable DNA methylation changes would suggest that these effects are independent of global alterations in DNA methylation levels.

**Figure 3:**
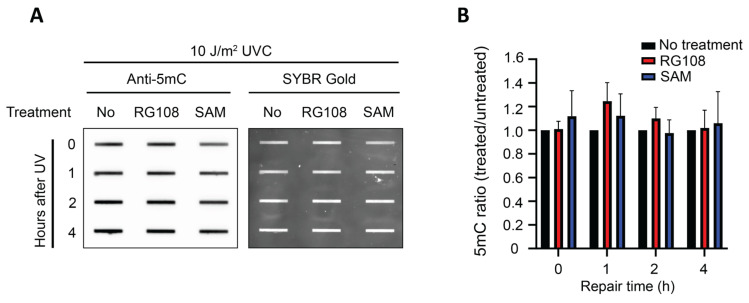
**Global 5mC levels following SAM and RG108 treatment and UVC irradiation.** (**A**) Representative immunoslot blot showing 5mC levels within 4 h in GM12878 cells treated with SAM (1000 µM) or RG108 (10 µM) for 24 h before UVC irradiation (10 J/m^2^). DNA was extracted at the indicated time points (0, 1, 2, and 4 h) post-irradiation and probed with an anti-5mC antibody. SYBR Gold staining was used as a loading control. (**B**) Quantification of global 5mC levels normalized to SYBR Gold signal and expressed relative to untreated controls at each time point. Data are shown as mean ± SEM from three biological experiments. Statistical analysis was performed using two-way ANOVA followed by multiple comparisons.

To address this, we quantified global 5mC levels under the same treatment and irradiation conditions used for the (6-4)PP formation and repair assays in GM12878 cells. Genomic DNA was extracted at 0, 1, 2, and 4 h post-irradiation and subjected to immunoslot blot assays using an anti-5mC antibody, with SYBR Gold staining as a loading control. Quantification of 5mC levels normalized to untreated revealed no statistically significant changes in 5mC levels across treatments and time points (Figure 3A,B). Together, these data indicate that the rapid suppression of UV lesion formation and the accelerated early (6-4)PP repair observed with SAM treatment occur in the absence of significant changes in global 5mC levels, suggesting that these effects are unlikely to be driven by large-scale alterations in DNA methylation.

### 3.4. Unaltered UV Lesion Repair Kinetics Following Post-Irradiation SAM or RG108 Treatment

Although SAM treatment prior to UV irradiation produced an apparent acceleration of early (6-4)PP repair, the concurrent reduction in initial damage levels raised the possibility that this effect was due to a reduced damage burden rather than enhanced intrinsic NER efficiency. To directly distinguish between these possibilities, we reversed the treatment sequence: cells were first exposed to UVC and then immediately treated with SAM or RG108. This design ensured equivalent initial damage levels across conditions, allowing repair kinetics to be assessed independently of initial lesion burden.

**Figure 4:**
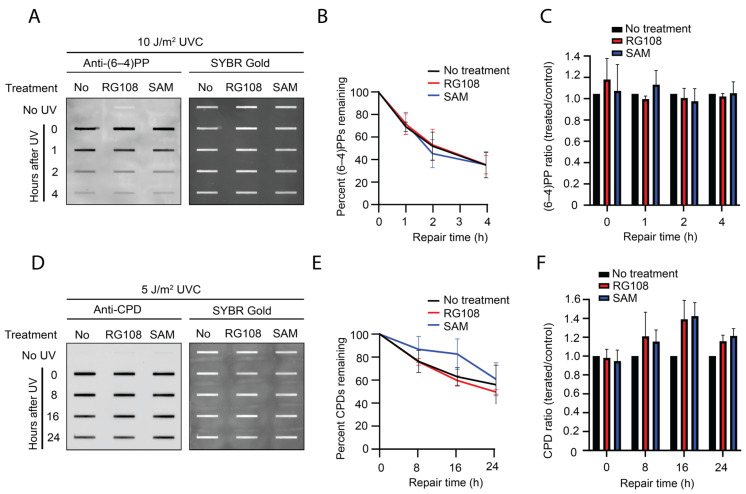
**(6-4)PP and CPD repair kinetics following post-irradiation SAM or RG108 treatment**. (**A**–**C**) Analysis of (6-4)PP repair kinetics in GM12878 cells irradiated with UVC (10 J/m^2^) and immediately treated with SAM (1000 µM) or RG108 (10 µM). Panels show representative immunoslot blots, quantification of repair kinetics, and (6-4)PP ratio (treated/untreated) across different time points. (**D**–**F**) Analysis of CPD repair kinetics exposed to UVC irradiation (5 J/m^2^) and immediately treated with SAM (1000 µM) or RG108 (10 µM). Quantification and data presentation are as described in (**A**–**C**). Data are presented as mean ± SEM from three biological experiments. Statistical significance was determined using two-way ANOVA followed by multiple-comparison testing. No statistically significant differences were detected between the treatment and untreated groups.

Under these conditions, there are no significant differences in the repair of either (6-4)PP or CPD in untreated, SAM-treated, and RG108-treated cells (Figure 4A–F). Damage levels decreased at a comparable rate across all treatment conditions in both UV lesion types (Figure 4B,E). Although a slight trend toward slower CPD removal was observed following SAM treatment, this effect did not reach statistical significance. Importantly, initial UV damage levels at the 0 h time point were similar when cells were treated with SAM or RG108 after UVC exposure (Figure 4C,F), confirming that any differences observed would reflect altered repair kinetics rather than differences in the initial damage formation.

These results indicate that neither SAM nor RG108 directly modulates NER when the DNA damage burden is at the same level. The findings also suggest that the accelerated early (6-4)PP repair observed following the SAM pretreatment (Figure 2) is primarily driven by reduced initial damage formation rather than by increased intrinsic NER activity. Under conditions of lower damage burden, the available NER machinery may be sufficient to remove lesions more rapidly, generating the appearance of accelerated repair without inherently altering NER capacity. Collectively, these findings suggest that SAM-mediated DNA methylation primarily influences UV lesion susceptibility rather than modulating NER capacity.

## 4. Discussion

In this study, we examined how modulation of DNA methylation using SAM, a universal methyl donor, and RG108, a DNMT inhibitor, influences UV-induced DNA damage formation and NER in human lymphoblastoid cells. Our findings reveal three principal insights. First, SAM rapidly suppresses UVC-induced formation of both (6-4)PPs and CPDs, with effects detectable within minutes. Second, although SAM pretreatment was associated with modestly accelerated early (6-4)PP repair, this apparent change reflects reduced initial damage burden rather than increased intrinsic NER capacity. These effects occur in the absence of detectable changes in global 5mC levels, suggesting a mechanism independent of large-scale alterations in DNA methylation. Third, neither SAM nor RG108 measurably alters NER kinetics when initial UV damage levels are constant. Together, these results indicate that SAM primarily modulates UV damage susceptibility, while overall NER capacity remains largely unaffected.

Our findings broaden the relationship between DNA methylation and UV-induced DNA damage formation. Several other studies have demonstrated that DNA methylation enhances CPD formation primarily under UVB, with UVC having little or no effect [23,53]. Yet, computational models exploring density functional theory techniques show that 5mC does not inherently lead to increases in CPDs after UV exposure [64]. In contrast, increased formation of (6-4)PPs at methylated cytosines has been reported after both UVB and UVC exposure [53]. Our results revealed a pronounced reduction in both (6-4)PP and CPD formation following SAM treatment, aligning closely with experiments performed by Leung et al. [52]. When taking sequence context into consideration, it should be noted that, unlike UVB, UVC has been shown to increase damage formation at certain sequence motifs while decreasing it at others [24].

While SAM serves as the methyl donor for DNMT-mediated cytosine methylation, elevated intracellular SAM can perturb methylation homeostasis through accumulation of S-adenosylhomocysteine (SAH), altered methyltransferase kinetics, and feedback inhibition [65]. Indeed, SAM treatments have been reported to induce both hypermethylation and hypomethylation in cancer cells [66]. Thus, the absence of global 5mC changes does not exclude localized alterations in DNA methylation patterns. Our slot blot assay measures global methylation levels, which may not be sensitive enough to detect minor changes in local methylation levels. Localized methylation patterns at specific loci may occur without producing detectable global changes. Local methylation patterns are still possible, but further studies need to be done to confirm a link between SAM treatment and lesion formation. Further nucleotide-resolution mapping of 5mC together with genome-wide UV damage profiling by using Damage-seq [67] or CPD-seq [68] would allow direct comparison of UV damage formation at methylated versus unmethylated cytosines under SAM or RG108 treatment [69]. Similarly, XR-seq [70] could be used to determine whether local methylation state influences NER efficiency at individual genomic loci. Beyond DNA methylation, SAM also directly affects histone methyltransferase activity. Changes in histone methylation, such as H3K4me3, H3K27me3, and H3K36me2/3 [71,72,73], can rapidly alter chromatin compaction, nucleosome positioning, and higher-order genome organization. Future profiling of histone modification landscapes and chromatin accessibility following SAM exposure will be necessary to determine whether histone methylation dynamics or chromatin compaction mediate the altered UV susceptibility observed in this study.

Since methylated cytosines within CPDs are prone to deamination, the epigenetic state influences both lesion formation and mutational outcome. Our data suggest that metabolic modulation of SAM levels can alter the initial burden of UV-induced damage without altering repair kinetics, thereby potentially shaping mutational risk at the earliest stage of damage induction. These findings reveal a previously unrecognized role for SAM in modulating UV damage susceptibility and highlight the importance of distinguishing formation-dependent from repair-dependent effects when interpreting NER kinetics. Because all of our experiments were performed in the GM12878 lymphoblast cell line, these findings should be interpreted within the context of only the GM12878 cell type, as chromatin organization and methylation landscapes can vary across cell types. UV photoproduct levels were quantified using immunoslot blot assays, which is a widely used approach for measuring (6-4)PP and CPD levels. However, this method detects lesions through antibody recognition and can be influenced by factors such as DNA loading, antibody affinity, and lesion accessibility within chromatin. Additional approaches, such as CPD-specific ELISA or locus-specific damage quantification methods, would help further validate whether the observed reduction reflects decreased lesion formation.

DNA methylation signatures have also gained increasing attention as biomarkers for cancer development. Methylation profiling of cell-free DNA (cfDNA) has emerged as a powerful non-invasive approach for early cancer detection and tissue-of-origin inference [74,75]. Because UV-induced lesion formation and mutation accumulation are influenced by methylation context, altered susceptibility to DNA damage may contribute to mutational signatures detectable in cfDNA-based diagnostic assays.

Future work aimed at identifying the molecular basis of SAM or RG108’s effect, mapping locus-specific methylation changes, and extending these observations to UVB and physiologically relevant cell types will provide deeper insights into the intersection of epigenetic modification and DNA damage repair.

## Data Availability

The original contributions presented in this study are included in the article. Further inquiries can be directed to the corresponding author.
